# Neural Substrates for Judgment of Self-Agency in Ambiguous Situations

**DOI:** 10.1371/journal.pone.0072267

**Published:** 2013-08-19

**Authors:** Hirokata Fukushima, Yurie Goto, Takaki Maeda, Motoichiro Kato, Satoshi Umeda

**Affiliations:** 1 Faculty of Sociology, Kansai University, Osaka, Japan; 2 Department of Psychology, Keio University, Tokyo, Japan; 3 Department of Neuropsychiatry, Keio University School of Medicine, Tokyo, Japan; University of Turin and the Italian Institute of Technology, Italy

## Abstract

The sense of agency is the attribution of oneself as the cause of one’s own actions and their effects. Accurate agency judgments are essential for adaptive behaviors in dynamic environments, especially in conditions of uncertainty. However, it is unclear how agency judgments are made in ambiguous situations where self-agency and non-self-agency are both possible. Agency attribution is thus thought to require higher-order neurocognitive processes that integrate several possibilities. Furthermore, neural activity specific to self-attribution, as compared with non-self-attribution, may reflect higher-order critical operations that contribute to constructions of self-consciousness. Based on these assumptions, the present study focused on agency judgments under ambiguous conditions and examined the neural correlates of this operation with functional magnetic resonance imaging. Participants performed a simple but demanding agency-judgment task, which required them to report on whether they attributed their own action as the cause of a visual stimulus change. The temporal discrepancy between the participant’s action and the visual events was adaptively set to be maximally ambiguous for each individual on a trial-by-trial basis. Comparison with results for a control condition revealed that the judgment of agency was associated with activity in lateral temporo-parietal areas, medial frontal areas, the dorsolateral prefrontal area, and frontal operculum/insula regions. However, most of these areas did not differentiate between self- and non-self-attribution. Instead, self-attribution was associated with activity in posterior midline areas, including the precuneus and posterior cingulate cortex. These results suggest that deliberate self-attribution of an external event is principally associated with activity in posterior midline structures, which is imperative for self-consciousness.

## Introduction

The “sense of agency”, an essential aspect of self-recognition, refers to the self-attribution of the cause of one’s own action, and its effects on the outside world [1]–[4]. The mechanisms underlying the sense of agency have primarily been proposed to involve an internal feed-forward model and a comparator mechanism, where the consequences of intentional actions are predicted by the internal feed-forward model, and then compared with the actual sensory feedback by the comparator mechanism [5]–[7]. The attribution of the event as self-generated is based on whether actual sensory input matches the prediction.

Importantly, the sense of agency can easily be ambiguous and uncertain in a dynamic environment. Consider a simple situation where an action–feedback discrepancy is the only source of information for an agency judgment. When the discrepancy between action and feedback is small, causation of an event can be easily attributed to the self, and when this discrepancy is large, causation can be attributed to external sources. However, there is high uncertainty when the magnitude of sensorimotor mismatch falls between these subjectively “small” and “large” amounts. As the environment can change dynamically, judgments about agency under uncertain conditions are important to guarantee adaptive and precise behaviors. The present study focused on the judgment of agency in such ambiguous situations.

Previous studies of the sense of agency have often employed the uncertain conditions described above in their experimental manipulations [8]–[11]. These studies provided ambiguous conditions to decrease the relative contribution of the sensorimotor cue to judgment of agency, with the main aim of examining the effects of other sources for the judgment. For example, individuals’ mental states, such as intentions, beliefs, and expectations, can also affect their sense of agency [12]–[15]. Additionally, multiple operations, such as implicit and explicit, and prospective and retrospective processes, have been proposed to build the sense of agency [3], [16], [17]. Despite these empirical and theoretical advances, however, the mechanism itself for agency judgment in purely ambiguous situations is still unclear.

It is considered that an ambiguous situation for agency judgment elicits a conflict between the perception of self-agency and non-self-agency. Thus, these potentially different judgments must be reconciled and integrated to produce a unitary sense of agency. This judgmental process may be generated by higher-order neurocognitive activity. Furthermore, it is assumed that the ability to differentiate between self- and non-self-agency is particularly important when making a judgment about agency attribution. That is, neural activity specific to self-attribution under ambiguous conditions, as compared with that involved in non-self-attribution, may reflect higher-order critical operations related to the construction of self-consciousness. On the basis of these assumptions, the aim of the present functional magnetic resonance imaging (fMRI) study was to elucidate the neural substrates underlying agency judgment in ambiguous situations. Particularly, we examined the activities selectively associated with *self*-attribution.

Uncertainty in judgment of agency can be produced in a complex manner; for instance, several factors such as environmental cues or prediction of the consequences possibly contradict each other to make the agency judgment difficult. However, the present study did not target such heterogeneous situations. Instead, as a first step of our approach, this study manipulated ambiguity simply in terms of the degree of action–feedback discrepancy. The participants were asked whether they attributed the self as a cause of change in a visual stimulus, which exhibited a variable degree of temporal discrepancy with their own actions. This type of task often includes visuo-proprioceptive correspondence between a subject’s body movement and visual feedback [10], [18]–[21], and is thus potentially contaminated with the sense of body-ownership being attributed to visual stimuli [17], [22]. In our task, the change in visual stimulus has no spatial correspondence to the participant’s own body movement, allowing the examination of a pure sense of agency [23], [24].

Furthermore, as the main feature of this study, an “adaptation method” was used on a trial-by-trial basis. This method determined the most ambiguous level of the task-relevant variable (i.e., temporal discrepancy) for each participant. Specifically, when a participant attributed self-agency to a certain magnitude of action–feedback delay, the delay in the next trial was extended so that the individual was less likely to make a self-attribution. Conversely, the delay was shortened when the subject attributed the movement in the previous trial to a “non-self” cause. We used this procedure because the temporal action–feedback discrepancy as a threshold of self/non-self discrimination depends on the individual, as does the amount of subjective ambiguity (e.g., [8], [24]–[27]). In previous studies, the sensorimotor gap has been parametrically varied and equally applied to each participant in each experiment (e.g., [9], [14], [28]). However, the adaptation method in the current study allowed us to provide participants the variables that were maximally ambiguous to each individual, to optimally strengthen the relative contribution of the higher-order judgment of agency.

Previous neuroimaging studies have suggested the involvement of several specific brain regions in the processes underlying the sense of agency. Activity in regions surrounding the temporo-parietal junction (TPJ), medial frontal lobe (consisting of the supplemental motor area (SMA) and anterior cingulate cortex (ACC)), the lateral frontal cortex (dorsolateral prefrontal cortex (DLPFC) and/or ventral premotor cortex), and insula cortex has been reported in studies of agency [10], [18]–[20], [28]–[30]. In addition, other regions such as the precuneus and posterior cingulate cortex (PCC), extrastriate body area (EBA), and cerebellum have been reported in agency studies [13], [18], [21], [31], [32]. In particular, previous studies have consistently implicated a region surrounding the TPJ, including the inferior parietal lobule (IPL) and posterior superior temporal sulcus (pSTS), reporting that increased activation in the TPJ and adjacent areas is reliably associated with decreases in the sense of agency [10], [19]–[21], [29], [33]. The TPJ is thought to receive signals indicating a mismatch between prediction and feedback, possibly indicating a need for a subsequent action to correct the mismatch [20], [21], [34]. However, this region is a rare case of consistency among research works. As an example of other areas, insula activity has been proposed to be associated with self-attribution in several studies [10], [19], [22], while other studies reported conflicting data [20], [27], [33] or failed to detect a contribution of the insula to agency processing [21], [33]. As such, the association of many brain areas to agency attribution remains unclear, and it is thus difficult to predict which region would contribute to agency processing when uncertainty is heightened.

In the current study, we presumed that the self-attribution in judgment of agency under uncertain conditions is mainly processed in the so-called midline cortical structure, which has recently been the subject of much research owing to its association with self-reflection and self-consciousness (e.g., [35], [36]). Particularly, we were interested in the posterior part of the medial region, which includes the precuneus, PCC and retrosplenial region. As in the case of frontal midline areas (i.e., ACC and adjacent areas), the involvement of the parietal midline areas has been reported in several previous studies of agency, but the functional contribution of these areas has not been examined in detail [10], [18]–[21], [29], [37], [38]. Although the frontal midline areas generally contribute to action and performance monitoring [39], [40], this function is not selective to self-behavior, but is shared with the perception of other individuals’ behavioral consequences [41]–[43]. On the other hand, even though the functional account of posterior midline areas is not yet mature, several findings have reported that the areas around the precuneus differentiate self- and other-attribution in agency judgment [18], [21], [29], [30] and are particularly biased to self-attribution [44]. Furthermore, these areas have anatomical and functional connectivity with several other areas, including the TPJ/IPL, insula, and SMA [45], [46], suggesting the existence of an integrative functional hub for agency-related areas. According to these points, we hypothesized that the posterior medial areas play a key role in the higher-order self-attribution of external events.

The present study investigated the neural activity associated with judgments of agency in a task where uncertainty was maximized. Our primary analysis focused on neural differentiation underlying self vs. non-self judgments, with the goal of uncovering the neural substrates specific to self-biased agency attribution during careful and reflective judgments. We predicted that the posterior medial regions, such as the precuneus and PCC, are essential for producing self-attribution of external events under ambiguous conditions.

## Methods

### Participants

Seventeen right-handed undergraduate Japanese students (6 males and 11 females, aged 19–22 years, mean ± standard deviation of 20.88±0.99 years) with no history of neurological, psychiatric, or ophthalmological disease participated in this study. All participants gave written informed consent before taking part in the study. The study was conducted in accordance with the Declaration of Helsinki and was approved by the Ethical Committee of Keio University (No. 09004).

### Tasks and Procedure

#### Apparatus

E-prime software (Psychology Software Tools, Inc., Pittsburgh, PA, USA) was used to present stimuli and record responses. A Victor LCD projector (60-Hz refresh rate) was used to back-project stimuli onto a screen (approximately 20°×32° visual angle/65×105 mm) with a mirror system mounted on the magnetic resonance imager head coil. The distance between participants’ eyes and the mirror was approximately 200 mm, with slight differences among participants due to variation in head size. Participants held response boxes (HHSC-2x4-C, Current Designs, Inc., PA, USA) in each hand and wore headphones (RTC2k, Resonance Technology Inc., CA, USA) in the scanner.

#### Agency-judgment trials

**Figure 1:**
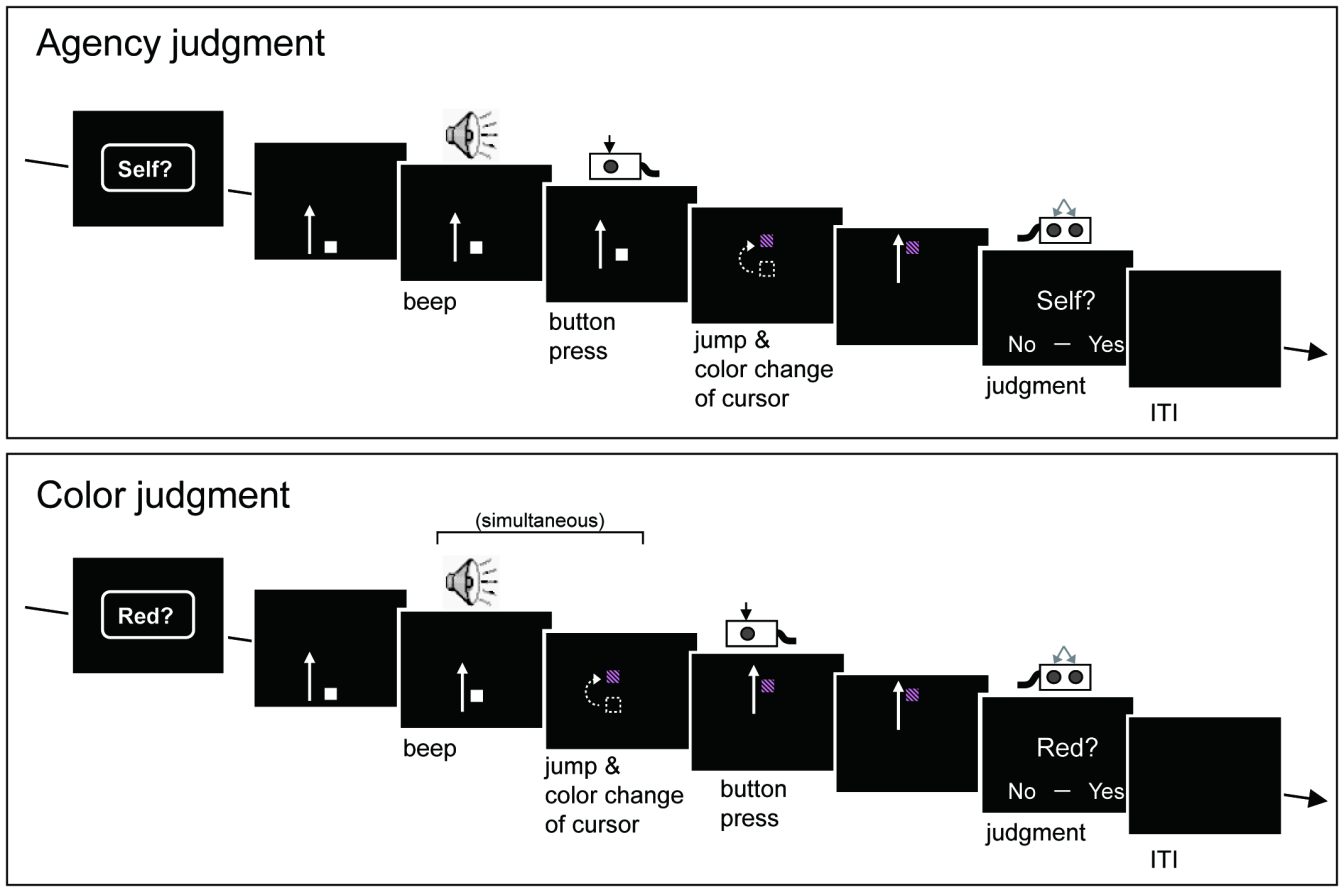
Task sequences. In each trial, a moving object changed its position and color at a moment proximate to the button-press response of a participant. Participants performed two types of judgment (agency-judgment and color-judgment) in a pseudo-random order. The agency-judgment condition required participants to report whether they felt they caused the change of a visual stimulus, while the color-judgment condition asked participants whether the color of the stimulus changed from gray to red. The timing of the stimulus change in the agency-judgment task and the color of the stimulus in the color-judgment task were adjusted on a trial-to-trial basis for each task. See the main text for details.

Participants completed two types of trials: agency-judgment and color-judgment (Fig. 1). In agency-judgment trials, participants were first prompted with a word (“Self?”) indicating that the next trial would require an agency judgment. This prompt lasted for 1 s and was followed by a black screen for 500 ms. A 3-mm gray square then appeared on a black background, emerging from the bottom of the screen and moving straight upward at a uniform speed (∼6.5 mm/s). A cue sound (1000-Hz pure tone with 100 ms duration) was presented ∼2 s (±100 ms) after the square appeared. Participants were instructed to press a key with their right index finger when they perceived a cue sound. Following a short delay (described in detail below) after the button press, the moving square on the monitor changed its coordinates (i.e., “jumped”) 6.8 mm upward, and changed color. The square object kept moving upward and disappeared into the upper outline of the display. The display then presented the words “Yes – No” to prompt the participant to respond on the basis of their judgment of self-agency, pressing a button to report whether they felt that the changes in the square’s position and color were caused by their own preceding action. Participants responded using the response box in their left hand, pressing a button with the index finger to indicate “yes”, and pressing a separate button with their middle finger to indicate “no”. The inter-trial interval varied between 3000 and 4500 ms in steps of 500 ms.

The temporal delay between the participant’s right-button press (cued by the tone) and the following alteration of the square was initially set to 400 ms following our previous study [23]. This delay was adjusted in each subsequent trial, depending on the participant’s judgment in the preceding agency-judgment trial. When participants reported “yes” in the previous trial (i.e., when they attributed the change in position of the square to self-agency), then the timing of the position change in the current trial was extended by 50 ms compared with the previous trial. If they reported “no” in the previous agency trial, the delay in the current trial was reduced by 50 ms. Using this procedure, in the first few trials, the length of the delay shifted towards a “borderline” of self and non-self judgment for each individual, and then fluctuated around that value. This means that participants were continually presented with a delay period that involved maximal uncertainty in judging self-agency. The average length (± standard deviation) of the delay (excluding the initial five trials) across all participants was 300.76 (±87.91) ms.

The color of the square (after changing) also varied across trials to match the visual properties of the stimuli with the color-judgment condition described below. The color was selected randomly from the set of possible colors in the color-judgment condition, independently of the participant’s performance.

#### Color-judgment trials

In color-judgment trials, participants judged the color of the square after its change. The color to be judged was adaptively set depending on the participant’s judgment in the former color trial, in the same manner as in the agency-judgment trial.

First, the word “Red?” was presented as a prompt, indicating that a color-judgment trial was about to begin. The task-sequence in color-judgment trials was identical to that in the agency-judgment trials, except for the timing of stimuli. In the color trials, a change in the square’s position and color occurred simultaneously with the cue sound (Fig. 1). This means that the change in the color and position of the square, and the cue sound, occurred *before* the button pressing, because participants were required to press the right button after the sound, as in the agency condition. The color-judgment trials were designed to completely eliminate any implicit sense of agency, which can arise when individuals observe a temporal sequence of their action and a subsequent change in visual stimuli. In the last part of the trial, a display presented the words “Yes – No”, prompting the participant to report whether the color of the square had changed to red. Participants responded with the left response box in the same way as in the agency condition.

The color of the square after changing was selected from a combination of red and blue. Decimal numbers in the 8-bit red-green-blue assignments were [250, 50, 100] for the reddest and [100, 50, 250] for the bluest color. The number of steps between these colors was the same as the number of steps of delay length in the agency condition. As with the delay length in the agency trial, the post-change color was adjusted depending on the preceding color-judgment trial. When a participant responded “yes” in the former trial, the color of the square was set one step toward blue. Conversely, when a participant reported “no” in the former trial, the color in the current trial was set one step toward red.

#### General procedure

After being instructed about both task conditions, participants completed ∼10 practice trials of each type outside the scanner. Immediately before the experimental session, participants performed 5–10 additional practice trials for each task in the scanner. In the experimental sessions, participants performed 132 trials in total (66 trials for each of the two task conditions), divided into three blocks. The order of the agency- and color-judgment trials was pseudo-randomized with the restriction that the same type of trial was not presented more than three times in succession. The assignment of response keys in the task was fixed among sessions and participants.

### fMRI Data Acquisition and Analysis

All images were acquired with a 3T Siemens Tim Trio scanner (Munich, Germany) having an eight-channel head coil. Scanning consisted of three experimental functional runs, with a high-resolution T1-weighted structural scan (3D MPRAGE with 1-mm isotropic resolution). Each functional run consisted of ∼240 whole-brain T2* weighted single-shot gradient-echo planar imaging (EPI) images, collected in an oblique axial orientation (TR of 2.35 s, TE of 30 ms, FA of 90 degrees, voxel size of 3.5 mm×3.5 mm×2 mm, 44 slices (descending), slice gap of 1 mm). The first three functional volumes were discarded to allow for equilibration of net magnetization. The structural scan was coregistered to the subject’s mean EPI image. The data were preprocessed and analyzed using the SPM8 software package (Wellcome Trust Centre for Neuroimaging, London, UK). Functional time images from each participant were spatially corrected for head movement, and temporally corrected for slice timing (using the middle slice acquired in time as a reference), spatially normalized to the Montreal Neurological Institute (MNI) template with a resample voxel size of 3.5 mm×3.5 mm×3.5 mm and spatially smoothed with a three-dimensional Gaussian filter (full width at half maximum of 8 mm). In addition, high-pass temporal filtering with a cut-off of 128 s was applied to remove low-frequency drift in the signal, and global changes were removed by proportional scaling.

Event-related effects were modeled using a 2×2 factorial design with factors of “condition” (agency-judgment/color-judgment) and participant’s “judgment” (yes/no). The first five trials for each condition were discarded from the fMRI (as well as behavioral) analysis, because initial performance was unstable while task variables (delay or color) were adaptively adjusted according to individual performance. For each session, regressors for a short period immediately after the participant’s action (time-locked to the timing of the right-button press, with a duration of 2 s) in the four conditions (agency/color conditions×yes/no judgments) were modeled by convolving the canonical hemodynamic response function (HRF) with its temporal and dispersion derivatives [47]. A 2-s period of HRF modeling was selected to detect reflective processing about action–-feedback events, which is assumed to take place later than automatic phasic responses at the event moment.

Statistical parametric maps were generated using the general linear model for each contrast of the *t* statistic on a voxel-by-voxel basis. Subsequent analyses of second-level group random effects were performed for the SPM contrast images of the first-level canonical HRF responses. A statistical threshold of *p*<0.001, uncorrected, with an extent threshold of 2 voxels for multiple spatial comparisons was used across the whole brain.

## Results

### Behavioral Results

Reaction times (RTs) in the judgment period (measured relative to the onset of the “yes–no” prompt) are given in Table 1. Repeated-measures analysis of variance (ANOVA) with the factors “condition” (agency-judgment/color-judgment) and “judgment” (yes/no) revealed no main effect of the condition, and no interaction between the condition and judgment. The ratio of “yes” to “no” responses was 1.06 (standard deviation of 0.22) for the agency trials and 0.93 (0.20) for the color trials, indicating that “yes” and “no” choices were reported in approximately equal numbers in both conditions. Paired t-tests revealed no significant differences between the two trial types (*p* = 0.11).

**Table 1:** Behavioral measures for each task condition.

	RT for "yes"	RT for "no"		"yes"/"no" ratio
Agency-judgment	521.05 (158.05)	462.72 (124.00)		0.51 (0.05)
Color-judgment	495.25 (160.06)	466.56 (129.38)		0.48 (0.08)

Note: Reaction times (RTs) in the judgment periods (in units of milliseconds) and the yes–no ratio of judgment for each condition are presented as means across participants. The values in parentheses are standard deviations.

### fMRI Results

#### Main effect of condition

**Figure 2:**
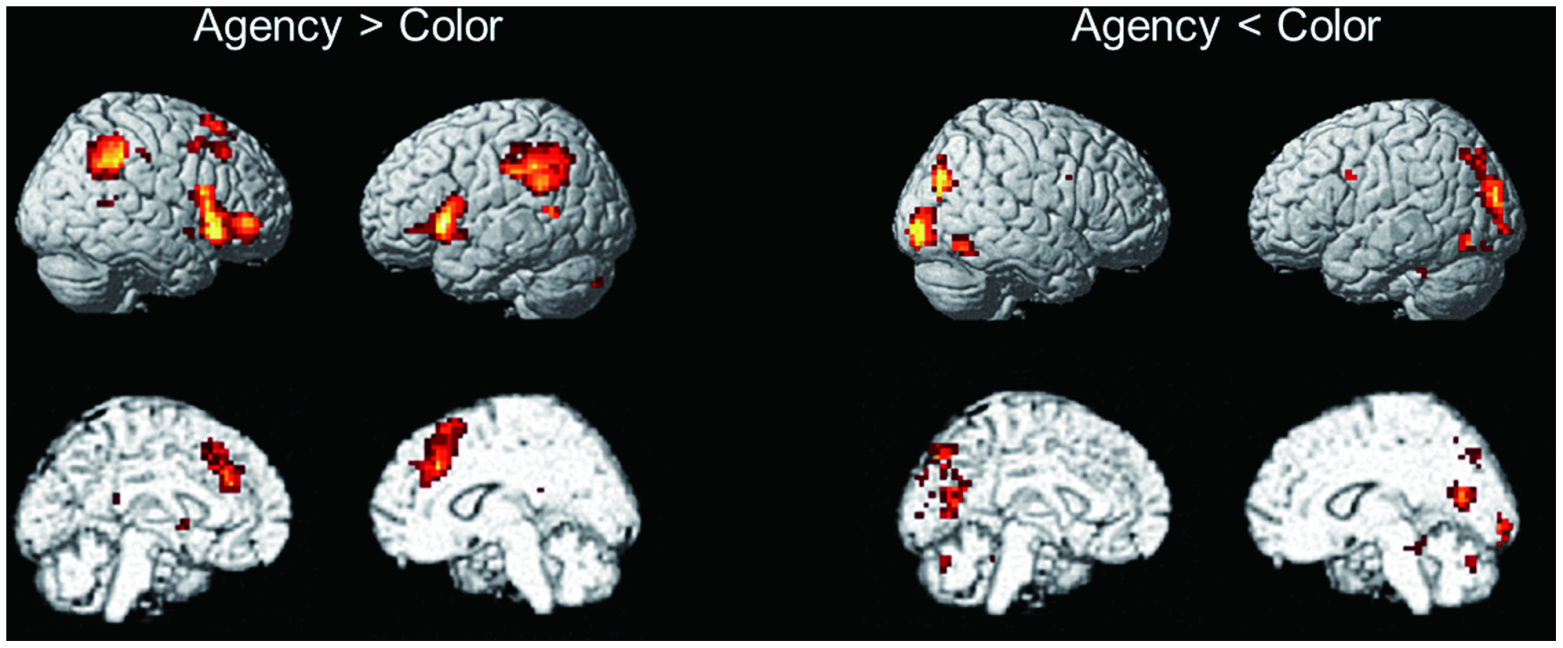
Regions associated with a between-condition difference. Left: A contrast of agency vs. color condition revealed a distributed neural network including the bilateral insula/operculum, IPL/TPJ, SMA/ACC, and right DLPFC. Right: A color vs. agency condition contrast revealed activity mainly among visual areas (*p*<0.001 uncorrected, with an extent threshold of 2 voxels).

To reveal the nature of the agency-judgment condition in the current study, the neural activity evoked in the agency trial was contrasted with that in the color-judgment trial. Increased neural activation for the agency condition relative to the color condition was found in the bilateral insula extending to the frontal operculum, IPL areas extending to the TPJ, the bilateral medial frontal gyrus including the SMA and dorsal anterior cingulate cortex, right middle frontal gyrus (DLPFC), and small portions of the cerebellum and PCC (Fig. 2; Table 2). In contrast, areas of stronger activation for the color condition relative to the agency condition were found predominantly in posterior regions of the brain, such as the lateral and medial occipital and inferior temporal cortex, as well as the cerebellum.

**Table 2:** Regions showing differential activity between task conditions (agency-judgment condition vs. color-judgment condition).

Location	Coordinates (MNI)			Z score	Voxels
	x	y	z		
*Agency condition – color condition*					
R IFG/Insula	41	25	−8	5.1	308
L Insula	−47	14	3	4.73	176
L Inferior parietal lobule	−57	−46	27	4.5	277
R Inferior parietal lobule	59	−53	38	4.34	188
L Superior temporal gyrus	−54	−53	10	4.23	16
R Medial frontal gyrus	6	28	38	4.21	218
R Middle frontal gyrus	38	28	45	3.92	36
R Superior temporal gyrus	45	7	−12	3.57	3
L Cerebellum	−26	−84	−40	3.56	4
R Postcentral gyrus	66	−28	45	3.34	7
L Posterior cingulate	−1	−39	17	3.27	2
*Color condition – Agency condition*					
L Precuneus	−19	−74	41	4.75	266
R Precuneus	41	−77	34	4.6	115
L Cerebellum	−29	−49	−22	4.26	50
R Fusiform gyrus	45	−60	−15	4.21	33
R Parahippocampal gyrus	34	−42	−12	3.99	40
R Cerebellum	3	−74	−29	3.69	10
L Inferior frontal gyrus	−43	7	31	3.61	8

To further clarify whether the between-condition difference shown above depended on the specific judgment (i.e., “yes” or “no”) made in each trial, activation in the agency-judgment trials compared with the color-judgment trials was examined for each judgment separately (i.e., agency vs. color condition comparison in trials with “yes” (or “no”) responses only; Table 3, Fig. S1). These contrasts revealed activity in regions similar to the regions for the main effect of the condition (i.e., the task comparison analyzing both “yes” and “no” trials together). Specifically, the between-condition comparison of “yes” trials revealed significant changes in all of the same regions except the PCC and post-central gyrus, which showed no significant changes. For “no” judgments, the same between-condition contrast revealed significant differences in most of the same regions as in the analysis of all trials, including the insula-operculum continuum, the anterior medial portions (ACC and SMA), and IPL. However, the size of significant voxels was diminished relative to the same contrast with “yes” judgments, and some regions, such as the DLPFC and cerebellum, did not exhibit significant activation in “no” trials.

**Table 3:** Regions showing differential activity between conditions for each type of judgment (“yes”/“no”).

Location	Coordinates (MNI)			Z score	Voxels
	x	y	z		
*Agency Yes – Color Yes*					
R Insula	48	11	17	5.2	232
L Inferior parietal lobule	−54	−46	45	4.87	289
R Inferior parietal lobule	55	−39	48	4.63	279
L Insula	−47	7	13	4.19	106
R Medial frontal gyrus	6	28	45	4.06	146
R Middle frontal gyrus	41	11	48	3.97	55
L Superior temporal gyrus	−57	−53	10	3.91	15
L Cerebellum	−26	−70	−33	3.8	12
*Agency No – Color No*					
R Superior frontal gyrus	13	14	62	4.03	26
R Cingulate gyrus	3	28	34	3.99	56
L Insula	−47	14	3	3.92	45
R Inferior frontal gyrus	38	42	−5	3.88	84
L Supramarginal gyrus	−61	−49	27	3.66	25
L Inferior parietal lobule	−50	−63	45	3.47	3
L Middle temporal gyrus	−47	0	−29	3.45	3
L Inferior parietal lobule	−57	−28	34	3.27	5
R Supramarginal gyrus	59	−42	38	3.22	13

#### Simple main effect of judgment for each condition

**Figure 3:**
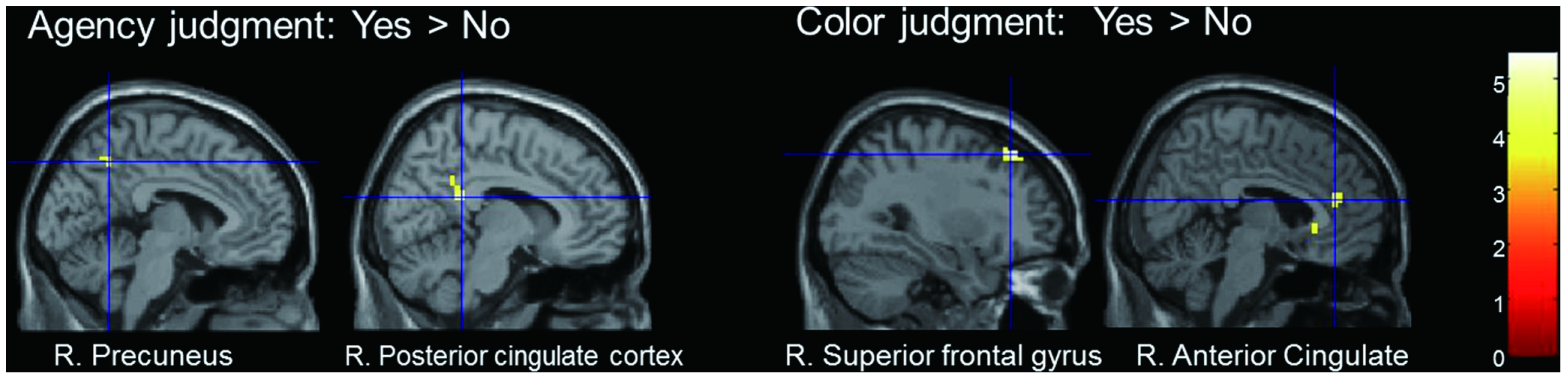
Activation for “Yes” versus “No” judgment in each condition. Left panels: A contrast of “yes” vs. “no” judgments for the agency-judgment condition revealed activity in posterior medial regions. Right panels: The same contrast as for the color-judgment condition revealed activation in the anterior part of the brain (*p*<0.001 uncorrected, with an extent threshold of 2 voxels).

The results above confirm that the agency-judgment condition mainly involved distributed cortical and subcortical networks that have been commonly implicated in previous agency studies (e.g., [29], [48]). Following this assessment, the main aim of the present study was to examine the brain areas playing an important role in self-attribution in the deliberate judgment of agency, which is thought to be reflected in the neural activity evoked in the “yes” trials of the agency condition. To this end, a significant simple main effect of judgment (i.e., “yes” vs. “no” trials) within each condition was analyzed separately (Table 4). For the agency condition, greater neural activity in the “yes” trials compared with the “no” trials was found in medial parietal areas (PCC and precuneus) and the right anterior insula as well as the inferior occipital cortex (Fig. 3). Moreover, the same “yes” vs. “no” contrast was calculated for the color condition in examining whether medial parietal activity was specific to the agency condition. As a result, the “yes” vs. “no” contrast for the color condition detected activation in different regions of the anterior cortex, such as the middle frontal gyrus and ACC. In addition, the “no” vs. “yes” contrast for each condition revealed activation in limited portions of cortical and subcortical regions (Table 4). No overlapping regions of activation were found between the agency- and color-judgment conditions in terms of the neural differentiation of yes/no judgments.

**Table 4:** Regions showing differential activity between “yes” and “no” judgment for each condition.

Location	Coordinates (MNI)			Z score	Voxels
	x	y	z		
*Agency Yes – No*					
R Posterior cingulate	10	−42	20	3.82	26
R Inferior occipital gyrus	38	−91	−8	4.05	13
L Cerebellum	−22	−84	−33	3.46	4
L Precuneus	−5	−56	48	3.45	4
L Superior temporal gyrus	−33	−60	27	3.24	2
L Precentral gyrus	−36	21	41	3.19	2
R Insula	38	18	13	3.17	2
*Agency No – Yes*					
R Postcentral gyrus	48	−25	62	3.23	5
L Cingulate gyrus	−8	0	31	3.58	2
L Cerebellum	−15	−32	−15	3.16	2
*Color Yes – No*					
R Middle frontal gyrus	31	32	52	4.01	22
R Anterior cingulate	6	39	17	3.76	14
Anterior corpus callosum	10	28	−1	4.22	9
R Superior frontal gyrus	17	35	41	3.63	2
*Color No – Yes*					
R Thalamus	13	−18	6	4.22	18
R Lentiform nucleus	20	4	13	4	12
L Lentiform nucleus	−26	−4	−5	3.26	6

## Discussion

In the present study, we sought to identify the neural substrates underlying judgments of self-agency under conditions of uncertainty. To this end, we adaptively set the task-relevant variables to be maximally ambiguous for each individual. We found that attributing self-agency to an external event in our task was associated with activity in posterior midline areas.

### Results of Basic Contrasts

Contrasts between the agency-judgment condition and color-judgment condition revealed significant neural activation in many of the cortical areas previously implicated in agency judgment (e.g., reviews in [29], [48]). These areas included regions around the TPJ and IPL, regions around the SMA and ACC, regions around the insula and frontal operculum, and the DLPFC. The between-condition differences within each type of judgment (i.e., conducting the contrast after separating “yes” and “no” judgment trials) revealed a similar pattern of activation changes. These results suggest that the experimental paradigm in this study, in which task difficulty was adaptively determined, was appropriate for examining agency-related processing.

However, the contrast between “yes” trials and “no” trials within both conditions revealed smaller areas of significant activity, in fewer brain regions. The results of the agency-judgment condition thus indicate that a large portion of the elicited neural activity did not differentiate between trials of self- and non-self-attribution. Perhaps this was because sensorimotor discrepancy was the only cue for judging agency in the current experiment; thus, automatic feelings of agency and reflective judgments of agency may have contributed equally to decisions in the “yes” (i.e., self-attribution) and “no” (non-self-attribution) trials. On the other hand, the contrast of “yes” vs. “no” trials for the agency condition would be expected to reflect the neural correlates of the higher-order processing, which may resolve the conflicting judgment of self- and other-agency in uncertain situations. We found small areas exhibiting significant activity in regard to this contrast, which were located in the medial parietal areas, including the precuneus and PCC. The result suggests that the posterior medial areas play an important role in judgment of agency in an ambiguous situation, particularly in the case of self-attribution. We consider that these regions are critical in the operation of self-ascription, rather than in the condition of uncertainty itself. This is because those activities were detected in terms of differentiation between self- and non-self-judgment, and because the degree of uncertainty should not be different among trials.

### Medial Parietal Areas Involved in Self-agency Attribution

Previous studies have occasionally reported the involvement of posterior medial regions in agency (e.g., [18]–[21], [29], [37], [38]). However, as for other regions of activation detected in the current study, it is currently unclear whether these areas contribute to the sense of agency. From the considerations discussed above, we suggest that the posterior medial regions may be suitable candidates for the cognitive process of binding external inputs with a representation of the self. Several structural and functional properties of the medial parietal regions support this interpretation, as described below.

First, anatomical connectivity studies have reported that posterior medial regions do not have direct connections with primary sensory cortices, but possess connections with several sensory (and sensorimotor) association areas [44]. Although previous findings are not entirely consistent, several functional connectivity studies have reported that medial posterior areas are associated with regions thought to be involved in agency, such as the TPJ/IPL, insula, and SMA [45]. These findings suggest that posterior medial areas may play an integrative role in the agency-related network involved in the cognitive process underlying judgments of agency. Furthermore, part of the precuneus is connected to the lateral PFC [45], [46], which is thought to be involved in higher-order cognitive processing (e.g., [49]). This finding supports the notion that the posterior medial area plays a critical role in the final attribution of agency, although it is still an open question as to which areas of the PFC and medial parietal are to be placed at a “higher” stage of the agency judgment.

Second, activity in medial parietal areas can also be interpreted according to their close association with consciousness and self-reflection [36], [50], [51]. The precuneus and PCC are closely connected to medial prefrontal regions, and have often been reported in studies of general self-reflection, as part of a network involved in mentalizing about the self and others [52]–[55]. Therefore, the role of medial parietal areas in self-attribution may also be related to self-reflection.

Alternatively, it is possible that the role of the medial parietal area in memory retrieval contributed to the judgment of agency in the current study. According to a recent theory, different components of the sense of agency can be characterized in terms of their temporal features, such as whether they are forward or backward processes (e.g., [3]). The automatic feeling of agency can be considered a type of predictive process, intrinsically relying on the internal feed-forward prediction of sensory consequences. Conversely, the cognitive judgment of agency can be regarded as a postdictive process; that is, interpreting the event with a *post-hoc* explanation. In accord with this notion, it is possible that the *post-hoc* judgment of agency, including the process of interest in this study, recruits memory processes involved in recall, and reflection on the action–feedback relationship occurred a short time earlier. The PCC is considered to be part of the Papez circuit, which is a network for memory encoding and retrieval [56], [57]. The precuneus is also reported to be active in memory retrieval [44]. Therefore, it is possible that these areas were activated by the postdictive self-attribution of the stimuli, involving memory components that are intrinsically private and self-related.

Previous studies reported inconsistent findings regarding the association between posterior medial areas and self- and/or non-self-attribution. Several studies suggested that medial parietal regions were associated with self-attribution [18], [21]. For example, Miele et al. [21] reported that the posterior precuneus and calcarine sulcus were more active when participants reported that they felt a sense of agency compared with trials in which they did not. In contrast, other studies reported that the precuneus and adjacent areas were more active when participants attributed the cause of an event to non-self-agency [29], [58]. Reviewing this inconsistency, Cavanna and Trimble [44] proposed an anterior–posterior segregation of the precuneus, and suggested that self-processing occurs in the anterior part (but see [29]). Interestingly, this region corresponds to the area exhibiting activity related to explicit self-attribution in the current study. Overall, the details of previous findings regarding the relationship between medial parietal regions and agency remain controversial. The present study extended previous findings, suggesting that these regions may be involved in conscious discrimination and attribution between self- and non-self agency. The present findings could be extended by combining our paradigm with tasks used in previous studies.

### Insula

In addition to medial parietal areas, areas of cortical activity differentiating between “yes” and “no” trials for the agency-judgment condition were also found in a small cluster in the right anterior insula. Brain regions frequently reported among agency studies, including the insula, are typically related with bodily processing. For example, areas around the inferior parietal lobe have been reported to integrate sensorimotor information to construct a body image, while dorsomedial frontal regions play a key role in performance monitoring [39], [59]–[61]. The insula is thought to be primarily involved in bodily processing by receiving interoceptive signals from visceral organs [62]–[64]. Thus, the insula appears to contribute to basic sensorimotor processing regarding agency. Indeed, we found the largest cluster of activity around the bilateral insula–operculum complex. At the same time, these areas did not clearly differentiate between trials resulting in self- and non-self-attribution. This may be because of the lack of variation in action–feedback discrepancy (sensorimotor cues) among trials.

However, the functional role of the tiny region of the anterior insula exhibiting significant activation in the self vs. non-self contrast remains unclear. A number of previous studies have reported that while the middle insula directly receives bodily input, the anterior portion of this region represents more abstract and subjective information of internal bodily states, generating self-reflection and self-consciousness from somatic signals (e.g., [53], [65], [66]). Therefore, anterior insula activation could represent a higher-order component of self-attribution, rather than low-level processes. Indeed, the insula is known to be active in processing information related to the self in various domains, such as facial recognition, action and emotion monitoring, and autobiographical memory [22], [67]–[70]. In light of the current results and previous findings, we tentatively propose that activity in the main part of the insula may subserve the somatosensory feeling of agency, while the anterior portion might also contribute to the explicit judgment of agency. These speculative predictions require further examination.

### TPJ/IPL

The temporo-parietal association areas (TPJ and IPL) are among the regions most frequently and robustly implicated in agency in previous studies (reviewed in [29]). Activity in these areas is reported to be positively correlated with action–feedback discrepancy, and to be active when individuals make an external (or non-self) attribution of an event [10], [13], [19], [20], [71].

For example, Miele et al. [21] demonstrated that the right TPJ responded to physical disturbance in visual feedback, rather than to subjective performance monitoring of participants. This and other related reports suggest, at least partially, the automaticity of TPJ involvement in detecting sensorimotor discrepancies [29], [30], [72]. According to these previous findings, we predicted that TPJ and adjacent areas would be more active during “no” judgment trials than during “yes” judgment trials in the current study. In the current task, however, the activity of these regions did not differentiate between “yes” and “no” judgments, as was the case for several other regions. That is, the TPJ was active not only when participants made non-self judgments, but also when they made self-attribution judgments (i.e., in “yes” trials). The participants in this study were almost always presented with a certain amount of action–feedback discrepancy. Thus, the TPJ may have been consistently activated by detecting the sensorimotor mismatch, irrespective of participants’ judgment. We propose that TPJ activity may have generated non-self attribution about an event across almost all trials, but this process might not be sufficient to make a final judgment of agency. Consequently, the final attribution is determined by activity in other areas, such as posterior medial regions. Note that we do not suggest that the TPJ (and other areas that did not differentiate self-other agency in this study) always functions in some “lower” stages in the agency judgment. Still, these areas might not play a critical role, at least in a situation where the ambiguous sensorimotor discrepancy is the only cue with which to judge agency.

### DLPFC

As this study assumed a higher-order integrative process in the difficult judgment of agency, one may predict that the PFC (particularly the dorsolateral part of it) would dominantly contribute to the judgment, because the DLPFC is thought to function in various types of conscious and explicit decision–related processing as a locus of supervisory executive function and meta-cognition [49], [73]. In fact, several investigations reported the contribution of the PFC in agency judgments [13], [28], [37], [74]. However, in the results of the present study, activities in the DLPFC and medial PFC were associated with agency judgment, but they did not differentiate self- and non-self-attribution of agency, as was the case in many other areas. That is, PFC regions did not appear to play an essential role in judging self-attribution in the current study. In our view, this finding is not necessarily inconsistent with popular assumptions of PFC functions as discussed below.

In reviewing the former agency studies, David et al. [48] proposed that the lateral PFC is associated with reflective and explicit judgment, rather than a pre-reflective and implicit feeling of agency. Their discussion was based on the framework of contrasting conscious vs. unconscious components. However, the present study does not fit with their scheme as it did not directly compare conscious and unconscious conditions. Instead, the current task required participants to undertake conscious processing in almost all trials by presenting similar delays that were difficult to judge. Therefore, this study does not contradict the possibility that the DLPFC is important in the conscious and reflective aspect of agency judgment.

Along another line, it is important to distinguish agency attribution from action monitoring, which is considered to be associated with PFC activity [49]. For instance, Miele et al. [21] conducted a tracking task with visual feedback involving disturbances, and revealed that the anterior PFC is activated during retrospective conscious evaluation of the task performance, rather than during execution of the task. Although their study suggested a supervisory role of the DLPFC in action control, again, this notion does not contradict our findings. We consider functional differentiation between the posterior medial regions and PFC, speculating that the former areas operate in the self-agency attribution of external stimuli, while the latter areas operate in monitoring an individual’s own action and possibly also contextual information.

### Yes vs. No Contrast in the Color-judgment Task

Compared with the agency-judgment condition, the color-judgment condition was associated with significant activity in a broad range of visual areas. However, contrasting neural activity between “yes” and “no” judgment trials for the color condition showed significant changes in only a small number of regions. These findings may reflect that stimuli resulting in “yes” and “no” judgments were almost identical, and that the neural substrates involved in both types of judgment were similar, as for the agency-judgment condition. The “yes” vs. “no” comparison for the color-judgment condition revealed activity in frontal portions of the cortex that were clearly different from the medial parietal regions implicated for the agency-judgment condition, as illustrated in Fig. 3. The functional role of the areas differentiating judgments for the color condition (i.e., the neural correlates of “red-attribution”), is currently unclear. We speculatively propose that activity in these areas may subserve general perceptual decisions about external stimuli, such as those relating to monitoring or attentional networks. In any case, it is clear that the regions of activation detected by the “yes” vs. “no” contrast for the agency-judgment condition and those for the color-judgment condition were markedly distinct, in spite of the similar task properties involved. The color-judgment condition was designed to match the perceptual (both visual and auditory) and motor components involved for the agency-judgment condition. In addition, it also matched other task properties of the agency-judgment condition, such as the amount of visual attention and task uncertainty involved, using an identical adaptive method. In fact, behavioral data (RTs and the “yes”/”no” ratios) did not differ between conditions, suggesting the validity of the color-judgment condition as a control for the agency-judgment condition. These issues and results lend further support to the notion that the brain regions discussed above contribute selectively to agency processing, rather than reflecting general information processing in perceptual judgment.

### Future Directions

In this final section, it is noted what the present study did not examine and left for future investigation. As the main feature of this study was to selectively focus on the situation of uncertainty, we did not measure the neural activities for “certain” conditions in which ambiguity for agency judgment hardly exists. To further support the current findings and clarify the neural substrates for the higher-order agency-attribution in an uncertain situation, it is important to directly compare ambiguous and non-ambiguous situations, or to parametrically vary the degree of ambiguity. Subjective ambiguity reported by participants should also be included as another variable of the examination. In addition, other factors such as the level of consciousness (e.g., comparing conscious vs. unconscious aspects of judgment) and contextual cues (e.g., an individual’s belief or prediction) can be included in experimental manipulations, to relate the current approach with several lines of former investigations [17], [48].

Finally, the current findings have implications for clinical research. A number of previous studies have investigated the cognitive aspects of schizophrenia, particularly the abnormal sense of agency [5], [75], [76]. Several recently proposed theories rely on multiple-factor frameworks and hypothesized atypical integration of agency-related processes in schizophrenia [3], [77]–[79]. We previously conducted a behavioral study of schizophrenia using a prototype version of the current task, reporting an aberrant sense of agency in patients with schizophrenia [23], [24]. The results of the present study suggest that the abnormal sense of agency in schizophrenia may be related to dysfunction in posterior midline cortex areas, including the precuneus and posterior cingulate cortex. Moreover, knowledge of the clinical aspects of the sense of agency may be extended by applying the adaptive paradigm used in the current study, which was found to be an appropriate method for elucidating the higher-order components of self-agency.

## Supporting Information

Figure S1 **Regions associated with a between-condition difference in each type of judgment (“yes” and “no”).** Left: The contrast of agency-judgment vs. color-judgment condition was calculated with the trials of “yes” judgments (left panels) and “no” judgments (right panels) separately. These analyses detected brain regions similar to those revealed in the simple comparison of agency vs. color condition shown in Fig. 3 (left) (p<0.001 uncorrected, with an extent threshold of 2 voxels).
(TIF)
