# Supplementary figures and images for: Neural Substrates for Judgment of Self-Agency in Ambiguous Situations
Source: PLoS One. 2013 Aug 19;8(8):e72267. doi: 10.1371/journal.pone.0072267 (PMC3747082; doi:10.1371/journal.pone.0072267)

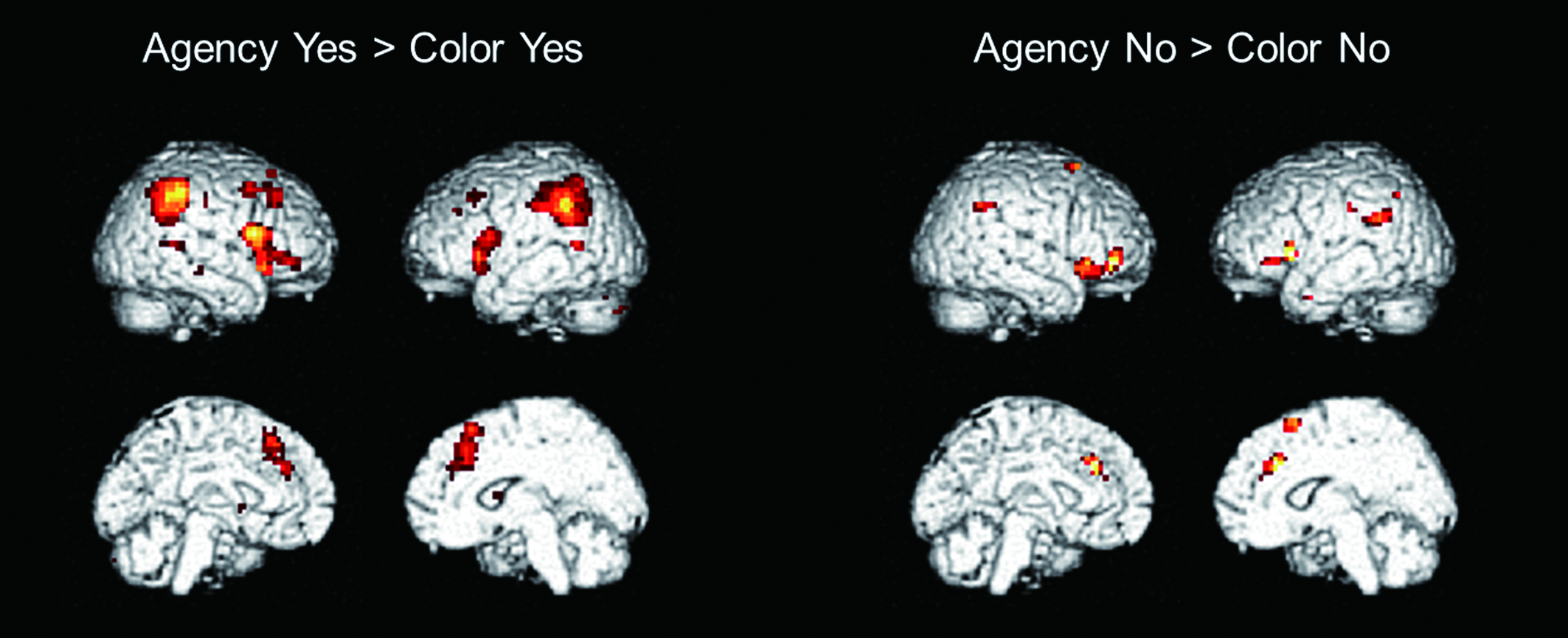

Supplement: Figure S1 — Regions associated with a between-condition difference in each type of judgment (“yes” and “no”). Left: The contrast of agency-judgment vs. color-judgment condition was calculated with the trials of “yes” judgments (left panels) and “no” judgments (right panels) separately. These analyses detected brain regions similar to those revealed in the simple comparison of agency vs. color condition shown in Fig. 3 (left) (p<0.001 uncorrected, with an extent threshold of 2 voxels). (TIF) [file pone.0072267.s001.tif]
